# Pancreas morphogenesis and homeostasis depends on tightly regulated Zeb1 levels in epithelial cells

**DOI:** 10.1038/s41420-021-00522-z

**Published:** 2021-06-11

**Authors:** María Lasierra Losada, Melissa Pauler, Niels Vandamme, Steven Goossens, Geert Berx, Moritz Leppkes, Harald Schuhwerk, Simone Brabletz, Thomas Brabletz, Marc P. Stemmler

**Affiliations:** 1 Department of Experimental Medicine 1, Nikolaus-Fiebiger Center for Molecular Medicine, Friedrich-Alexander University of Erlangen-Nürnberg, Erlangen, Germany; 2 Molecular and Cellular Oncology Laboratory, Department of Biomedical Molecular Biology, Ghent University, Ghent, Belgium; 3 VIB-UGent Center for Inflammation Research, Ghent, Belgium; 4 Cancer Research Institute Ghent (CRIG), Ghent, Belgium; 5 Department of Diagnostic Sciences, Ghent University and University Hospital, Ghent, Belgium; 6 Department of Medicine 1, Translational Research Center and Kussmaul Campus for Medical Research, Deutsches Zentrum Immuntherapie (DZI), University of Erlangen-Nürnberg, Erlangen, Germany; 7 Comprehensive Cancer Center Erlangen-EMN, Erlangen University Hospital, Friedrich-Alexander University Erlangen-Nürnberg, Erlangen, Germany

**Keywords:** Organogenesis, Type 2 diabetes

## Abstract

The pancreas is comprised of exocrine and endocrine compartments releasing digestive enzymes into the duodenum and regulating blood glucose levels by insulin and glucagon release. Tissue homeostasis is depending on transcription factor networks, involving Ptf1α, Ngn3, Nkx6.1, and Sox9, which are already activated during organogenesis. However, proper organ function is challenged by diets of high sugar and fat content, increasing the risk of type 2 diabetes and other disorders. A detailed understanding of processes that are important for homeostasis and are impaired during type 2 diabetes is lacking. Here, we show that Zeb1—a transcription factor known for its pivotal role in epithelial-mesenchymal transition, cell plasticity, and metastasis in cancer—is expressed at low levels in epithelial cells of the pancreas and is crucial for organogenesis and pancreas function. Loss of *Zeb1* in these cells result in an increase of islet mass, impaired glucose tolerance, and sensitizes to develop liver and pancreas steatosis during diabetes and obesity. Interestingly, moderate overexpression of *Zeb1* results in severe pancreas agenesis and lethality after birth, due to islet insufficiency and lack of acinar structures. We show that *Zeb1* induction interferes with proper differentiation, cell survival, and proliferation during pancreas formation, due to deregulated expression of endocrine-specific transcription factors. In summary, our analysis suggests a novel role of Zeb1 for homeostasis in epithelial cells that is indispensable for pancreas morphogenesis and proper organ function involving a tight regulation of Zeb1 expression.

## Introduction

Proper pancreas organ function and homeostasis is highly challenged by the life style and diets of modern societies, leading to continuously raising disorders like type 2 diabetes, pancreatitis, and pancreatic cancer^1–4^. The pancreas is organized in an exocrine compartment, producing and releasing digestive enzymes into the intestine, and an endocrine compartment, i.e., the islets of Langerhans^2^. Endocrine cells regulate glucose concentrations in the blood by insulin and glucagon releasing β- and α-cells, respectively^5,6^. Although endocrine cells are very adaptable to different metabolic demands, the pancreas is vulnerable to continuous diets with high carbohydrates and fat content^2^. Consequently, type 2 diabetes is constantly rising, which is characterized by reduction of the insulin-producing β-cell mass, insulin production/secretion, loss of sensitivity to insulin, liver and pancreas steatosis and inflammation. To identify more efficient treatment options a more detailed understanding of pancreas organogenesis and function is needed.

During development the pancreas in mice is specified at E9.0 by budding from the ventral foregut endoderm into dorsal and ventral parts that eventually fuse at E12.5^1,5,6^. *Pdx1* expression is initiated in multiprogenitor cells that are specified by local repression of Shh, involving activin, retinoic acid, FGF, and BMP signals^1,5,6^. Pdx1, Ptf1α, and Sox9 are crucial for pancreas organogenesis and loss of any of these factors results in agenesis or hypoplasia^7–10^. During primary transition until E12.5 FGF10 signaling from the underlying mesoderm promotes *Ptf1a* and *Sox9* expression to form tip (acini progenitors) and trunk cells (endocrine and duct progenitors)^11–13^. Ptf1α and Nkx6.1/Nkx6.2 specify tip and trunk cells, respectively, by repressing each other’s expression, orchestrated by the Notch pathway^5,12,14–16^. Secondary transition is initiated at E12.5 and lasts until E16.5, involving continued branching morphogenesis and specification of tip cells into acini by E15.5 and of endocrine progenitors by delamination of individual Ngn3+ cells from the monolayer epithelium of the trunk to form islets^1,11,16–19^. During the second transition Ptf1α, Pdx1 and Sox9 become restricted to acini, islets and ducts, respectively^6^.

Endocrine cell delamination requires activation of epithelial-mesenchymal transition (EMT)^20^, a program that is activated also during tumorigenesis by EMT transcription factors including Snail, Slug, and Zeb1/2. Zeb1 is well known for regulating tumorigenesis, metastasis, and therapy resistance^21–27^. Activation of *Zeb1* induces a transient loss of epithelial characteristics and a gain of mesenchymal traits enabling cell motility and malignancy^24,26,28^. We recently identified that, although Zeb1 is almost undetectable in normal epithelia and tumor cells before EMT, an intact *Zeb1* allele promotes initiation and progression of precancerous lesions of the pancreas (PanINs)^22^. During embryogenesis and in adult tissues, *Zeb1* is mainly confined to neuronal and mesodermal lineages, whereas epithelia display only weak or no Zeb1 expression^29,30^. *Zeb1* knockout embryos show severe skeletal defects, impaired neural tube closure, and die perinataly^29,31^. Zeb1 is involved in the cell-type specification, tissue function, and regeneration, including muscle, bone, and melanocyte differentiation^32–35^. However, not much is known about Zeb1 function in epithelia. Here, we analyzed the role of Zeb1 during pancreas formation and homeostasis using loss- and gain of function approaches in mice. We found that Zeb1 is involved in the maintenance of tissue homeostasis and protects the pancreas from accelerated tissue damage during metabolic disorders, like diabetes mellitus. Moreover, our data indicate that Zeb1 levels need to be tightly regulated to form and maintain a pancreas with appropriate acinar to islet ratios and to avoid tissue damage.

## Results

### Depletion of *Zeb1* in the embryonic pancreas results in increased organ weight

**Fig. 1:**
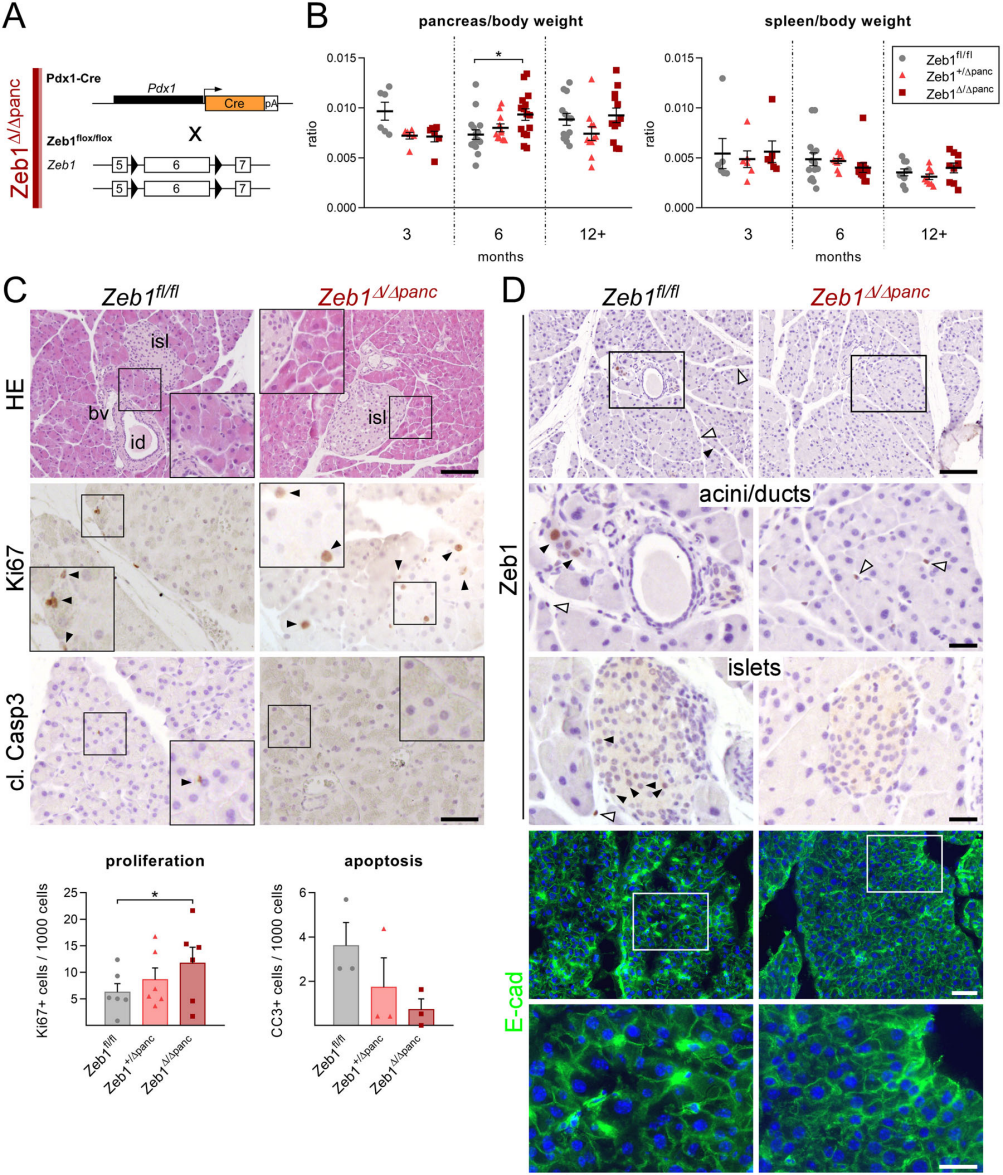
Depletion of *Zeb1* in the epithelium of the embryonic pancreas results in increased pancreas mass in adult mice. **A** Strategy of pancreas-specific *Zeb1*-depletion in acinar, ductal, and endocrine lineages by combining *Pdx1*-Cre and *Zeb1*^*flox*^ alleles in *Zeb1*^Δ/Δ*panc*^ mice. **B** Analysis of pancreas to body weight and spleen to body weight ratios in 3, 6 and 12+-month-old mice, demonstrating a pancreas-specific increase in organ size at 6 months of age. *n* = 6 (3 months), *n* = 11–16 (6 months), *n* = 11–13 (12 + months). **C** Histological (HE) and anti-Ki67/anti-cleaved Caspase 3 immunhistochemical stainings on paraffin-embedded pancreas sections of 6-month-old mice with summary graphs of quantification of proliferating and apoptotic cell fractions. Arrowheads indicate examples of positively stained cells. bv, blood vessel; id, intralobular duct; isl, islet of Langerhans. *n* = 6 (Ki67), *n* = 3 (cl. Casp. 3). Scale bars, 100 µm (HE) and 50 µm (IHC). **D** anti-Zeb1 immunohistochemistry and anti-E-cadherin (E-cad) immunofluorescence staining, showing proper Zeb1 loss in epithelial cells in acini and islets of Langerhans of 6-month-old *Zeb1*^Δ/Δ*panc*^ pancreata without affecting the expression of E-cad as one major Zeb1 target. Arrowheads and open arrowheads indicate Zeb1-positive epithelial and mesenchymal cells, respectively. Scale bars, 100 µm (upper), 25 µm (lower IHC), 50 µm (upper) and 25 µm (lower IF). Statistical significance was determined by two-way ANOVA and indicated if significance was reached. *p = 0.0194 (**B**); *p = 0.0280 (**C**).

We explored Zeb1 expression in non-mesenchymal cells by using the zygotic *Zeb1* knockout^29^. Besides strong expression of Zeb1 in the brain, neural tube, and mesoderm-derived tissues, we detected weak expression in the pancreas epithelial compartment, which was lost in *Zeb1* knockout embryos (Suppl. Fig. S1A). To specifically target *Zeb1* in these cells we used conditional gene ablation by *Pdx1*-Cre^29,36^ starting from E8.5. Efficient, but not always uniform recombination was observed with the mTmG Cre-reporter allele (Suppl. Fig. S1B)^37^ and *Pdx1*-Cre mediated *Zeb1* depletion (hereafter *Zeb1*^*Δ/Δpanc*^) was restricted to the pancreas epithelium, including acini, ducts, and islets (Suppl. Fig. S1B). No obvious malformation or atrophy was found upon *Zeb1* loss (Fig. 1A). However, mutant mice displayed an increase in pancreas mass, albeit significant only at six months of age due to high individual variability (Fig. 1B, left). Such alterations were not observed in other organs (Fig. 1B, right). At six months heterozygous mice also showed a trending increase in pancreas mass (Fig. 1B). Tissue architecture and abundance of the exocrine compartment (acini and intralobular ducts) and endocrine islets of Langerhans were not affected (Fig. 1C). We detected an increase in proliferation by Ki67 and a reduced, but not yet significant, level of apoptosis by cleaved Caspase 3 immunohistochemistry in pancreata of *Zeb1*^*∆/∆panc*^ animals. In adult pancreata epithelial-specific Zeb1 expression was confined to only individual acinar and islet cells, but was absent in *Zeb1*-deficient mice. Yet, no effect of *Zeb1* depletion on protein expression of *bona fide* Zeb1 target genes, like E-cadherin (E-cad), was detected (Fig. 1D).

These results show that Zeb1 is expressed at low levels in pancreas epithelial lineages during development and in adult mice and that *Zeb1* depletion results in increased organ weight likely supported by imbalanced proliferation and apoptosis rates.

### *Zeb1* deficiency affects normal function of the endocrine pancreas

**Fig. 2:**
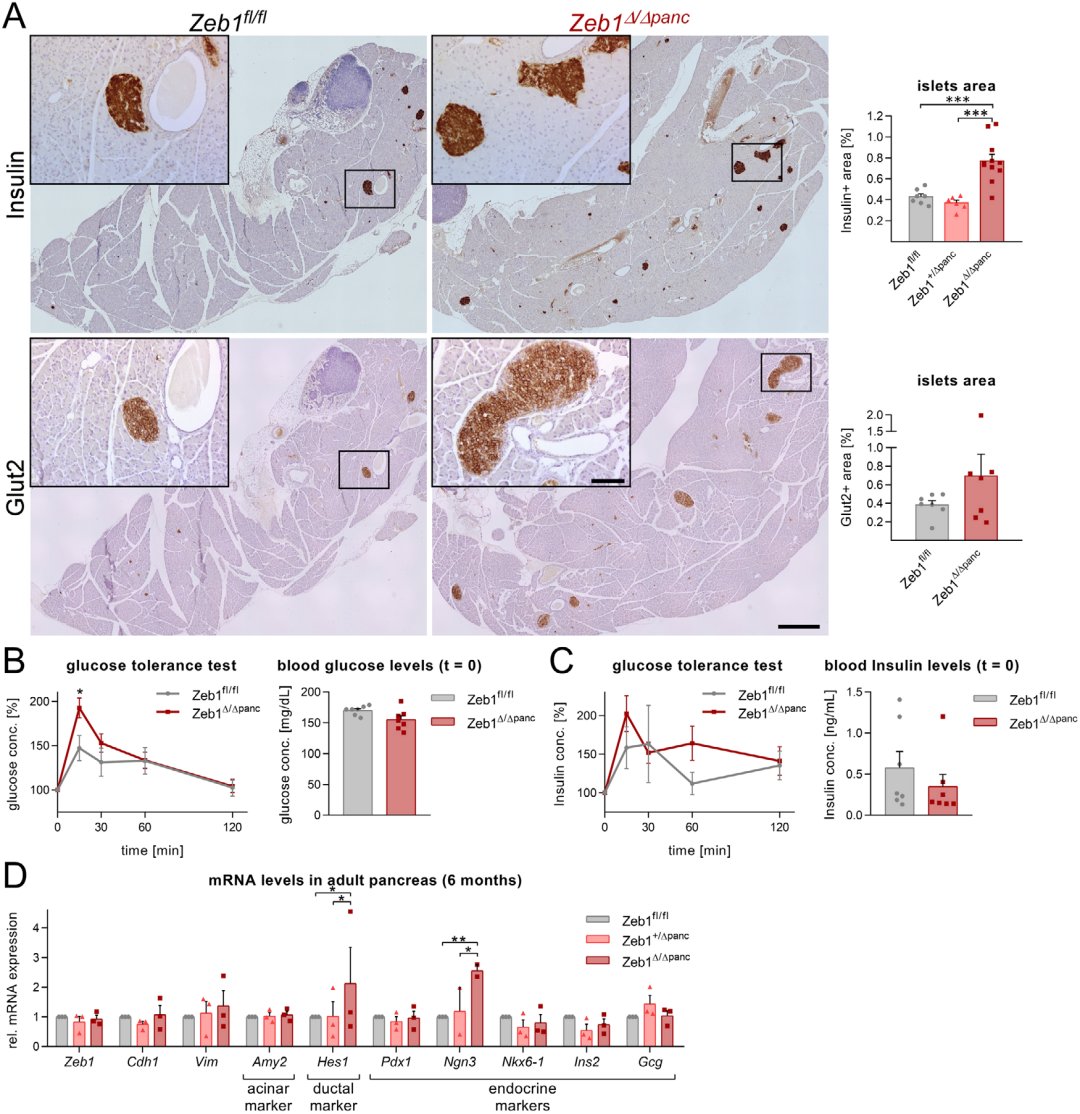
Zeb1 in the pancreas is crucial for regulating islet mass and function. **A** Immunhistochemical staining of islets of Langerhans by anti-Insulin and anti-Glut2 in pancreata of 6-month-old mice, revealing an increase in islet mass upon Zeb1 depletion. *n* = 6–8 (Insulin), *n* = 7 (Glut2). Scale bars, 500 µm and 100 µm (inset). **B**, **C** Intraperitoneal glucose tolerance test (GTT) in *Zeb1*^Δ/Δ*panc*^ and *Zeb1*^*fl/fl*^ littermates at 6 months of age and measurement of relative glucose (**B**, left) and insulin levels in venous blood over time (**C**, left), as well as absolute glucose (**B**, right) and insulin levels (**C**, right) before injection (*t* = 0). *n* = 7. **D** qRT-PCR analysis of *Zeb1*^Δ/Δ*panc*^, *Zeb1*^+/Δ*panc*^ and *Zeb1*^*fl/fl*^ pancreas mRNA at 6 months detecting EMT markers and lineage-specific genes (*Amy2*, acinar; *Hes1*, ductal; *Pdx1*, *Ngn3*, *Nkx6-1*, *Ins2*, *Gcg*, endocrine), demonstrating that specifically endocrine marker genes are differentially expressed in *Zeb1*^Δ/Δ*panc*^ mutant mice. *n* = 3. Statistical significance was determined by two-way ANOVA (qRT-PCR), one-way ANOVA (Insulin, Glut2), unpaired two-tailed multiple (GTT) or single Student’s *t*-test (GTT, *t* = 0) and indicated if significance was reached. ***p = 0.0003, *p* < 0.0001 (**A**); *p = 0.0197 (**B**); **p = 0.0086; *p = 0.0407, *p* = 0.0452, *p* = 0.0425 (**D**).

Combined with an increase in organ mass *Zeb1*^*∆/∆panc*^ mice showed elevated Insulin-positive islet β-cell areas (Fig. 2A). To address whether this increase in islet mass had also functional impact on blood glucose regulation we subjected *Zeb1*^*fl/fl*^ and *Zeb1*^Δ/∆*panc*^ littermates to an intraperitoneal glucose tolerance test (GTT)^38^. As expected, intraperitoneal (i.p.) injection of 2 g/kg body weight glucose raised blood glucose levels to ~150% in control mice (Fig. 2B, gray line), which declined to pre-injection levels within 120 min. In contrast, glucose levels increased up to 200% in *Zeb1*^∆/∆*panc*^ littermates (Fig. 2B, red line) and remained higher than those of control mice until 60 min post glucose injection, indicating that acute endocrine function was impaired upon *Zeb1* loss. Interestingly, Insulin concentrations also showed a higher, but still not significant peak in *Zeb1*^∆/∆*panc*^ mice, whereas they trend to be slightly reduced before fasting and GTT (*t* = 0; Fig. 2C) as well as in untreated, non-fasting mice (Suppl. Fig. S2A). The low-affinity glucose transporter, Glut2 regulates membrane depolarization and Insulin granule fusion with the plasma membrane in β-cells; one key step in blood glucose level sensing^39^. In accordance with the increased area of islets we detected also a trend towards increased Glut2-positive areas in *Zeb1*^∆/∆*panc*^ mice but no deregulation within individual cells or islets (Fig. 2A). Pancreas bulk mRNA analysis revealed no significant decrease in *Zeb1* transcript levels and no changes in Zeb1-regulated targets, like E-cad (*Cdh1*) and Vimentin (*Vim*) (Fig. 2D), owing to the major contribution of *Zeb1* expression in the pancreas by mesenchymal cells not targeted by *Pdx1*-Cre. A specific lineage marker for acinar cells, Amylase (*Amy2*), was unchanged, whereas the ductal marker and Notch target *Hes1* was increased in *Zeb1*^*∆/∆panc*^ pancreata. Although the endocrine lineage and differentiation transcription factor *Pdx1* was not altered, two downstream transcription factors for β-cell differentiation, were either significantly upregulated (*Ngn3*) or tended to be reduced (*Nkx6-1*) in mutants (Fig. 2D).

In summary, our findings show that cell composition is shifted in the *Zeb1*^Δ/Δ*panc*^ pancreata towards an increase of islet β-cell mass, combined with altered endocrine marker gene expression likely causing systemic impairment on sensing/processing of blood glucose levels.

### *Zeb1* depletion reveals metabolic changes in diabetic mice

**Fig. 3:**
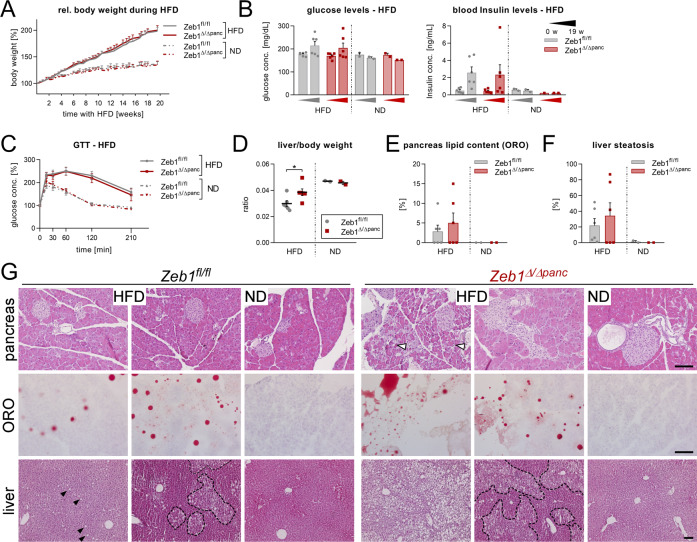
*Zeb1* loss results in systemic metabolic changes accompanied by increased lipid deposit in the pancreas and liver upon challenging the endocrine pancreas. **A** Induction of type 2 diabetes by high-fat diet (HFD) over 19 weeks leads to rapid and similar body weight gain in *Zeb1*^Δ/Δ*panc*^ and *Zeb1*^*fl/fl*^ littermates compared to normal chow diet (ND). *n* = 11–12 (HFD), *n* = 4 (ND). **B** Measurement of blood glucose and insulin levels before and after HFD shows no difference between the genotypes. *n* = 6 (HFD), *n* = 2 (ND). **C** Intraperitoneal GTT in mice after the 19-week HFD. *n* = 9 (HFD), *n* = 4 (ND). **D** Measurement of the liver to body weight ratios indicate a significant increase in *Zeb1*^Δ/Δ*panc*^ liver mass compared to *Zeb1*^*fl/fl*^ littermates after HFD. *n* = 6 (HFD), *n* = 2 (ND). **E**, **F** Percentage of area that shows lipid content deposit by oil red O (ORO) staining on cryosections (**E**) and liver steatosis on HE sections (**F**) of *Zeb1*^Δ/Δ*panc*^ and *Zeb1*^*fl/fl*^ mice after HFD. *n* = 6 (HFD), *n* = 2 (ND). **G** HE staining on paraffin sections of the pancreas (upper panel) and liver (lower panel) of *Zeb1*^Δ/Δ*panc*^ and *Zeb1*^*fl/fl*^ mice after HFD or ND as well as detection of lipid droplets in the pancreas by ORO on cryosections (middle panel), indicating a more severe phenotype in HFD-fed *Zeb1*^Δ/Δ*panc*^ mice. Note, that pancreas and liver HE sections are taken from the same mouse, whereas ORO stained sections are taken from a different individual. Open arrowheads indicate detection of altered pancreas histology and arrowheads and dashed lines highlight liver steatosis. The absence of a dashed line indicator in the *Zeb1*^Δ/Δ*panc*^ liver (HFD) refers to the absence of normal liver tissue, whereas livers from ND-fed mice are devoid of liver steatosis. Scale bars, 100 µm. Statistical significance was determined by two-way ANOVA and indicated if significance was reached. *p = 0.030.

To discriminate Zeb1 function between endocrine and exocrine compartments we challenged the pancreas by induction of type 2 diabetes^40^ and chronic pancreatitis (CP). Mice subjected to high-fat diet (HFD) for 19 weeks developed type 2 diabetes, dramatically gained body weight (Fig. 3A, Suppl. Fig. S2B), and displayed elevated blood glucose levels by the trend in comparison to normal diet (ND)-fed mice. Moreover, a two to eightfold but not yet significant increase in steady-state Insulin levels was detected (Fig. 3B, Suppl. Fig. S2C–E), suggesting insulin resistance. In general, males were more susceptible to gain body weight than females and individuals show varying consequential effects. Although no apparent differences in body weight, blood glucose, and insulin levels were observed between the genotypes, only *Zeb1*^Δ/Δ*panc*^ animals showed a tendency of retaining high glucose levels after the short period of fasting prior to GTT (Suppl. Fig. S2D). Upon increasing baseline glucose levels (*t* = 0) HFD-fed diabetic *Zeb1*^Δ/Δ*panc*^ mice displayed no change in the glucose peak at 15 min after glucose injection and the diabetes-dependent impairment of glucose clearance (Fig. 3C, Suppl. Fig. S2F). Importantly, HFD-fed *Zeb1*^Δ/Δ*panc*^ mice exhibited increased liver weight (Fig. 3D, Suppl. Fig. S2G). Despite high variability, we consistently found higher fractions of them displaying lipid deposition in the pancreas (≥40%, three out of six vs. one out of six mice) and apparent liver steatosis (≥40%, three out of six vs. two out of six mice; Fig. 3E–G, Suppl. Fig. S2G, H). Of note, *Zeb1*^Δ/Δ*panc*^ males showed trending increased liver steatosis, but decreased pancreas lipid content in comparison to their control littermates, whereas *Zeb1*^Δ/Δ*panc*^ females showed inverted effects (Suppl. Fig. S2I).

To challenge the exocrine compartment in *Zeb1*^Δ/Δ*panc*^ mice, we performed hydrodynamic gene delivery of a constitutive *Il17a* expression vector to induce pancreatitis^41^. CP was detected in *Zeb1*^*fl/fl*^ and *Zeb1*^Δ/Δ*panc*^ individuals 15 days post injection, accompanied by massive tissue damage, especially of acinar cells, infiltration of CD45+ hematopoietic cells, and fibrosis without major differences (Suppl. Fig. S3).

In conclusion, *Zeb1* deficiency mainly affects the endocrine compartment, leading to altered glucose and insulin levels that result in systemic effects by exacerbating disease pathology of type 2 diabetes including accelerated liver steatosis.

### Moderate induction of *Zeb1* in the pancreas causes agenesis

**Fig. 4:**
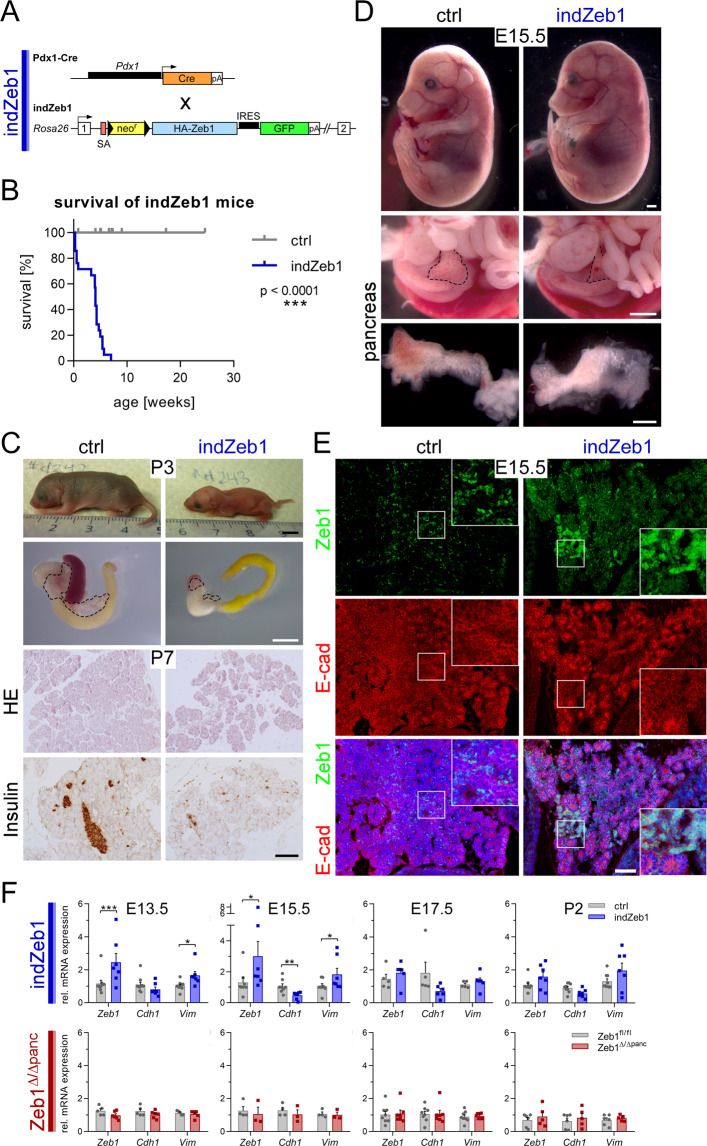
Moderate experimental *Zeb1* induction in the pancreas causes a moribund phenotype and pancreas agenesis. **A** Strategy to generate indZeb1 mice. A targeted allele of the *Rosa26* locus provides ectopic *Zeb1* expression under *Rosa26 cis*-regulatory elements that is unleashed by Cre activity to delete a loxP flanked *PGK*-neo-3xpA cassette. An internal ribosomal entry site (IRES) allows simultaneous expression of eGFP in recombined cells. Zeb1 expression was directed to the developing pancreas from E8.5 onwards utilizing the *Pdx1*-Cre allele. **B** Kaplan–Meyer plot showing the moribund phenotype of indZeb1 pups displayed already in early postnatal stages with only a few individuals surviving more than 4 weeks. *n* = 11 (ctrl), *n* = 20 (indZeb1). **C** Analysis of pups at P3 demonstrating severe growth retardation and pancreas agenesis. Scale bar, 5 mm (upper) and 100 µm (lower panels). **D** Isolated indZeb1 embryos at E15.5 show no developmental defects and a pancreas that is comparable in size of ctrls. Some mutant embryos display cyst formation or hemorrhage. Scale bars, 1 mm (upper and middle) and 500 µm (lower panel). **E** Immunofluorescence staining of Zeb1 and E-cadherin (E-cad) in the pancreas on sagittal cryosections of ctrl and indZeb1 embryos at E15.5, demonstrating that Zeb1 is moderately induced throughout the pancreas epithelium with patches of more increased expression (inset). E-cad expression is reduced in indZeb1 embryos compared to ctrls. Embryos are oriented anterior to the top and dorsal to the right. Nuclei are stained with DAPI (blue). Scale bar, 100 µm. **F** Detection of transcript levels of *Zeb1*, *Cdh1* (E-cad), and *Vim* in embryonic pancreata between E13.5 and P2, demonstrating two to threefold induction of *Zeb1* in early stages of indZeb1 specimens, combined with slight *Cdh1* reduction and *Vim* upregulation, whereas no change was observed in *Zeb1*^*Δ/Δpanc*^ individuals. *n* = 7–11 (E13.5, indZeb1), *n* = 5 (E13.5, *Zeb1*^*Δ/Δpanc*^); *n* = 7–9 (E15.5, indZeb1), *n* = 3–4 (E15.5, *Zeb1*^*Δ/Δpanc*^); *n* = 5–6 (E17.5, indZeb1), *n* = 7–8 (E17.5, *Zeb1*^*Δ/Δpanc*^); *n* = 7–9 (P2, indZeb1), *n* = 5–6 (P2, *Zeb1*^*Δ/Δpanc*^). Statistical significance was determined by Log-rank test (survival) or Student’s *t*-test (qRT-PCR) and indicated if significance was reached. ***p < 0.0001 (**B**); ***p < 0.001; **p = 0.007; *p = 0.012, *p* = 0.022, *p* = 0.080 (**F**).

*Zeb1* deletion in the pancreas affects pancreas homeostasis. To experimentally increase Zeb1 levels we made use of the indZeb1 knock-in allele (*R26-Zeb1*^*tg*^), harboring Zeb1-HA cDNA driven by the *Rosa26* locus^42^ activated by *Pdx1*-Cre in indZeb1 mice (Fig. 4A). To our surprise, these mice died shortly after birth showing severe growth retardation with only a few individuals reaching 2–7 weeks of age (Fig. 4B, C), due to pancreas agenesis to variable extend and sporadic cyst formation. This observation was combined with more severe reduction of the islet mass (Fig. 4C). To discriminate whether moderate elevation of *Zeb1* expression impaired pancreas specification or affected cell survival later, we analyzed E15.5 embryos, when the secondary transition was almost completed in control embryos. Although the size of the embryonic pancreas was slightly reduced, all indZeb1 embryos displayed a clearly discernible pancreas with proper gross morphology but included sporadic cystic or blood-filled structures (Fig. 4D). *Zeb1* overexpression was confirmed by immunofluorescence staining, showing expression at the detection limit, with a few patches of more robust expression (Fig. 4E and inset). We observed a slight global reduction in the expression of the Zeb1 target E-cad with almost negative patches of higher Zeb1 levels in mutant embryos (Fig. 4D). mRNA analysis of pancreata from embryos and pups confirmed a moderate 1.5 and 3-fold increase in *Zeb1* transcripts resulting in lower *Cdh1* and higher *Vim* levels in indZeb1, whereas *Zeb1* depletion had no robust effect (Fig. 4F).

In summary, we established mice with an approximately twofold ectopic *Zeb1* expression in pancreas epithelial cells that is incompatible with proper pancreas organogenesis resulting in agenesis at birth.

### Proper pancreas morphogenesis in indZeb1 mice is impaired during secondary transition

**Fig. 5:**
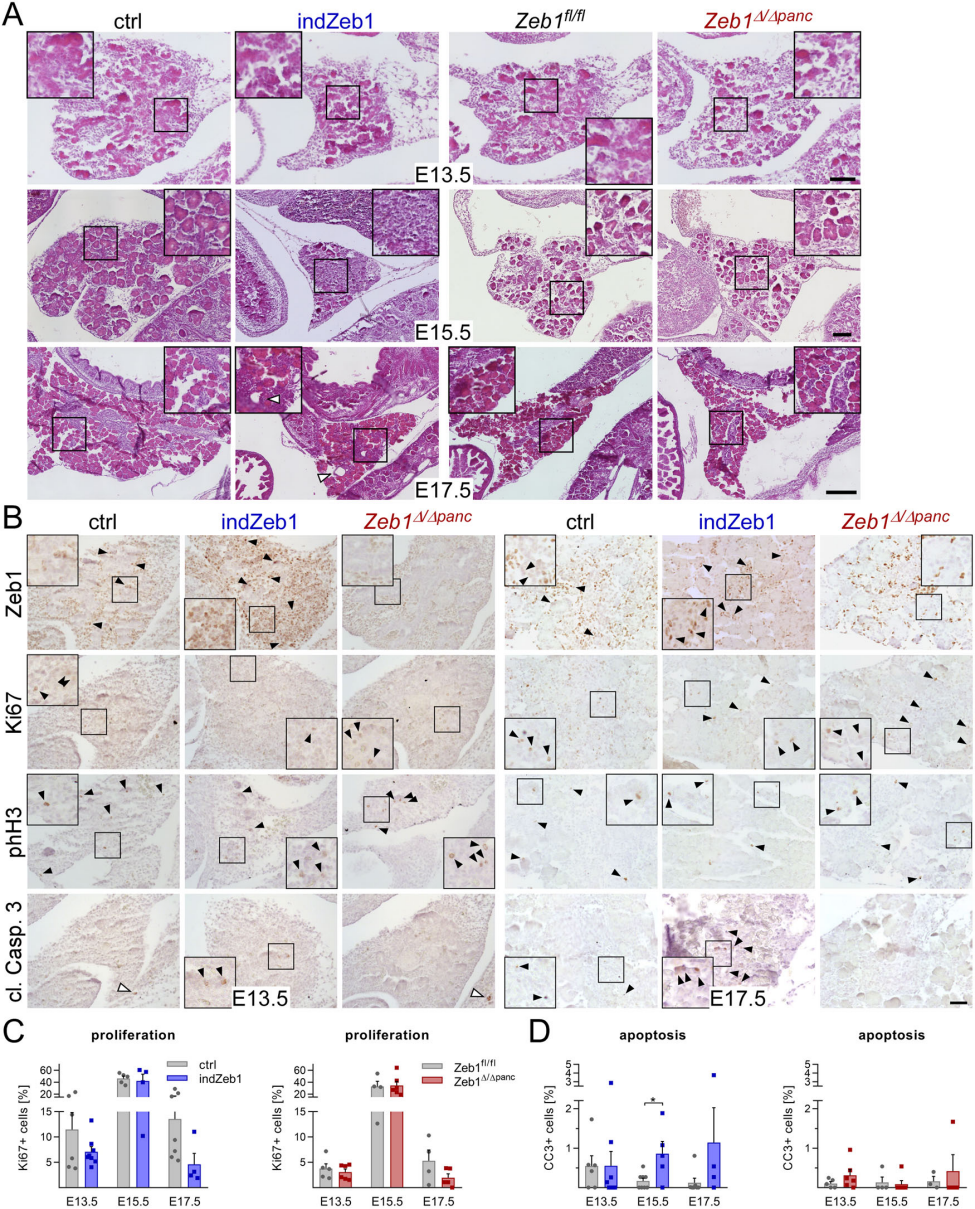
Pancreas organogenesis is impaired after primary transition in indZeb1 embryos. **A** HE staining of sagittal paraffin sections of embryos of ctrl, indZeb1, *Zeb1*^*fl/fl*^, and *Zeb1*^Δ/Δ*panc*^ genotypes between E13.5 and E17.5 as indicated. Whereas no morphological alterations were detected upon *Zeb1* depletion indZeb1 embryos show specific defects that include the presence of undefined cell aggregates/clusters that do not show separation into tip/trunk cells or acini/duct/islet progenitors as present in control littermates (E13.5, E15.5), reduction of the expansion of the pancreas in some embryos (all stages) and appearance of cyst-like structures (E17.5, open arrowheads). Scale bars, 100 µm (top and middle panels), 250 µm (bottom panel). **B** Analysis of proliferation (anti-Ki67, anti-phH3) and apoptosis (anti-cleaved Caspase 3) and Zeb1 by immunohistochemical staining on consecutive sections of ctrl, indZeb1, and *Zeb1*^*Δ/Δpanc*^ embryos at E13.5 and E17.5. Arrowheads point to examples of positive cells in the epithelial compartment. Note, that apoptotic cells are very rare and not found on every section or only in other organs (open arrowheads). Scale bar, 50 µm. **C**, **D** Quantification of Ki67- (**C**) and cl. Casp. 3-positive cells (**D**) in indZeb1 and *Zeb1*^*Δ/Δpanc*^ embryos in comparison to their corresponding control littermates at indicated stages. Proliferation in indZeb1 individuals is reduced at E13.5 and E17.5, while apoptosis is increased at E15.5 and E17.5. No difference was observed upon *Zeb1* depletion. *n* = 4–8 (indZeb1), *n* = 4–6 (*Zeb1*^*Δ/Δpanc*^). All sections are oriented anterior to the top and dorsal to the right. Scale bar, 50 µm. Statistical significance was determined by Student’s *t*-test and indicated if significance was reached. *p = 0.020.

To identify how pancreas formation is compromised by elevated Zeb1, we analyzed key developmental stages after completion of the primary transition (E13.5), during (E15.5), and after the secondary transition (E17.5) on a cellular level in indZeb1 and *Zeb1*^Δ/Δ*panc*^ embryos. Histological analysis confirmed that the size of the pancreas of indZeb1 was affected mainly during the secondary transition at E15.5 and E17.5 to a varying extend (Fig. 5A). No apparent defects were observed in *Zeb1*^Δ/Δ*panc*^ pancreata throughout organogenesis (Fig. 5A). In Zeb1-proficient control (ctrl) and *Zeb1*^*fl/fl*^ littermates at E13.5 cellular differentiation into tip and trunk cells was identified by HE staining (Fig. 5A, top panel). In contrast, this proper morphological separation was not present in indZeb1 specimens. Here, we observed more undifferentiated clusters of cells that became more prominent at E15.5 and acinar morphogenesis was only initiated in some areas of the pancreas (Fig. 5A, middle panel). Moreover, separation into individual lobes that were present at E15.5 in ctrl and *Zeb1*^*fl/fl*^ embryos was clearly lacking in indZeb1 individuals. These developmental defects were more pronounced at E17.5, when the pancreas was smaller in indZeb1 embryos (Fig. 5A, lower panel, arrowheads), although proper acinar and duct structures were found in less affected areas. Reduction in organ size was reflected by a trend to decreased proliferation and increased apoptosis (Fig. 5B–D), whereas it was unchanged in *Zeb1*^Δ/Δ*panc*^ embryos.

Our findings indicate that ectopic Zeb1 expression is interfering with secondary transition and blocks proper lineage specification leading to an accumulation of undifferentiated cells combined with indications of impaired proliferation and increased apoptotic rates.

### Elevated *Zeb1* expression in the embryonic pancreas interferes with proper endocrine differentiation

**Fig. 6:**
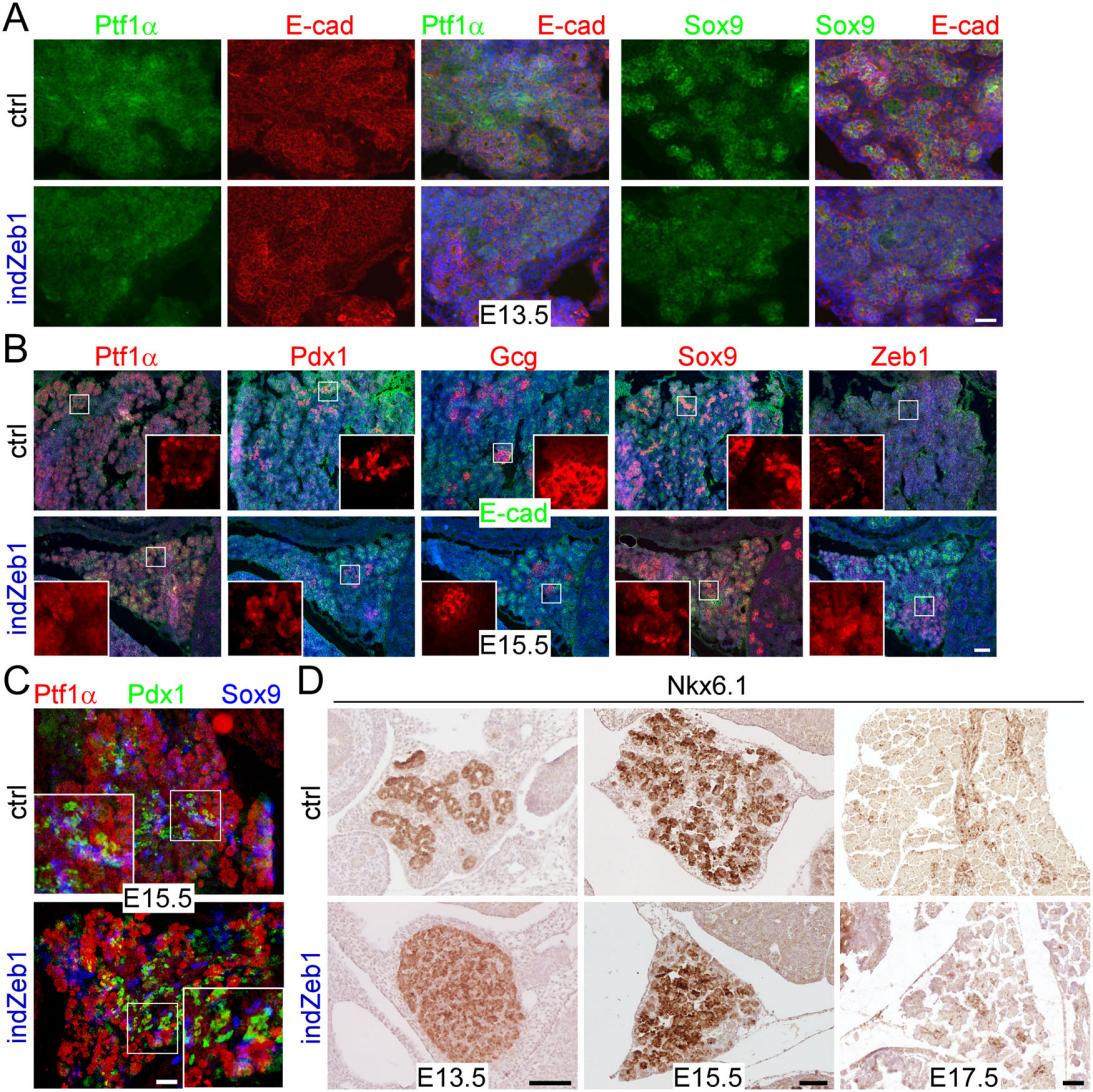
Ectopic *Zeb1* activation results in reduction of progenitors of the endocrine lineage. **A** Immunofluorescence labeling of Ptf1α (tip) and Sox9 (trunk) on sagittal cryosections of indZeb1 and ctrl embryos at E13.5, showing Ptf1α and Sox9 staining in both genotypes, indicating that tip and trunk cell specification is unaffected in indZeb1 embryos. However, a slightly reduced number of Ptf1α and Sox9 positive cells is detected in indZeb1 embryos. Anti-E-cadherin staining (red) was used to visualize tissue architecture. Nuclei are labeled with DAPI (blue). Scale bar, 50 µm. **B** Immunofluorescence labeling of Ptf1α (acinar), Pdx1 (endocrine), Glucagon (Gcg, islet β-cell progenitors), and Sox9 (duct) on sagittal cryosections of indZeb1 and ctrl embryos at E15.5. Markers of all lineages are detected, however, specifically, the number of Pdx1 and Gcg-positive cells are reduced in indZeb1 mutant embryos, indicating a specific loss of endocrine progenitors. Anti-Zeb1 and anti-E-cad labeling were used to visualize cells with ectopic expression and tissue architecture, respectively. Insets show red channel only of the boxed region. Nuclei are labeled with DAPI (blue). Scale bar, 100 µm. **C** Pseudo-overlay of consecutive sagittal cryosections, individually stained for either Ptf1α (acinar), Pdx1 (endocrine), or Sox9 (duct lineage). False colors were used to visualize the topological organization of the different cell lineages in the pancreas of indZeb1 and ctrl embryos at E15.5. In indZeb1 the topological tissue organization is maintained, displaying Ptf1α-positive acinar structures on the outside with patches of Pdx1-positive endocrine cells and Sox9-positive ducts in the center of the tissue. Scale bar, 100 µm. **D** Immunohistochemical staining of Nkx6.1 in indZeb1 and ctrl embryos between E13.5 and E17.5. Nkx6.1 labels tip/trunk and acini/duct/endocrine progenitor cells after primary transition and becomes confined to endocrine cells at later stages in ctrl embryos. In indZeb1 specifically, undefined unstructured accumulation of cells are all stained positive for Nkx6.1, indicating a block in proper lineage segregation. Scale bar, 100 µm. All sections are oriented anterior to the top and dorsal to the right.

Next, we checked for lineage marker gene expression, i.e., Ptf1α for tip/acinar cells, Sox9 for trunk/duct cells, and Pdx1 and Glucagon for endocrine/α-cells. At E13.5 we found uniform expression of Ptf1α with a preference to acinar/tip structures and Sox9 labeled trunk/duct/endocrine progenitors in ctrl embryos (Fig. 6A, top panel). indZeb1 embryos also displayed Ptf1α expression, but slightly weaker, whereas Sox9 was less confined, indicating that lineage segregation was delayed or inhibited upon *Zeb1* induction (Fig. 6A, bottom panel). At the end of the secondary transition at E15.5 Ptf1α properly decorated acinar structures, Pdx1 and Glucagon were found in more centrally located endocrine derivatives and Sox9 in ductal cells of ctrl embryos (Fig. 6B, top panel, Suppl. Fig. S4A). Upon *Zeb1* overexpression acinar and duct cells were still identified by Ptf1α and Sox9, respectively (Fig. 5B, bottom panel, Suppl. Fig. S4A). However, we noticed a substantial reduction of Pdx1 and Glucagon positive cells, indicating a reduction of the endocrine lineage. This was most apparent in patches of higher ectopic Zeb1 levels, in which also E-cad was reduced, but without altering Ptf1α and Sox9 expression (Fig. 6B, Suppl. Fig. S4A). Areas with milder defects displayed proper tissue architecture with proper marker gene expression (Fig. 6C, Suppl. Fig. S4B). Nkx6.1 is a key transcription factor for lineage specification in multiprogenitor cells in early stages and for islet β-cell formation later during organogenesis^43^. In E13.5 ctrl pancreata Nkx6.1 labeled distinct epithelial trunk and tip cell clusters and was continuously expressed until E15.5 (Fig. 6D). In contrast, indZeb1 embryos showed uniform expression of Nkx6.1 in more undifferentiated cell clusters at E13.5 and E15.5 (Fig. 6D). At E17.5 the number of Nkx6.1-positive cells was largely reduced in indZeb1 compared to ctrl littermates (Fig. 6D).

This analysis demonstrates that pancreas cell differentiation and morphogenesis are sabotaged by moderate *Zeb1* overexpression, mainly affecting the endocrine lineage.

### *Zeb1* induction reduces expression of miR-200 family members that are regulators of β-cell function

**Fig. 7:**
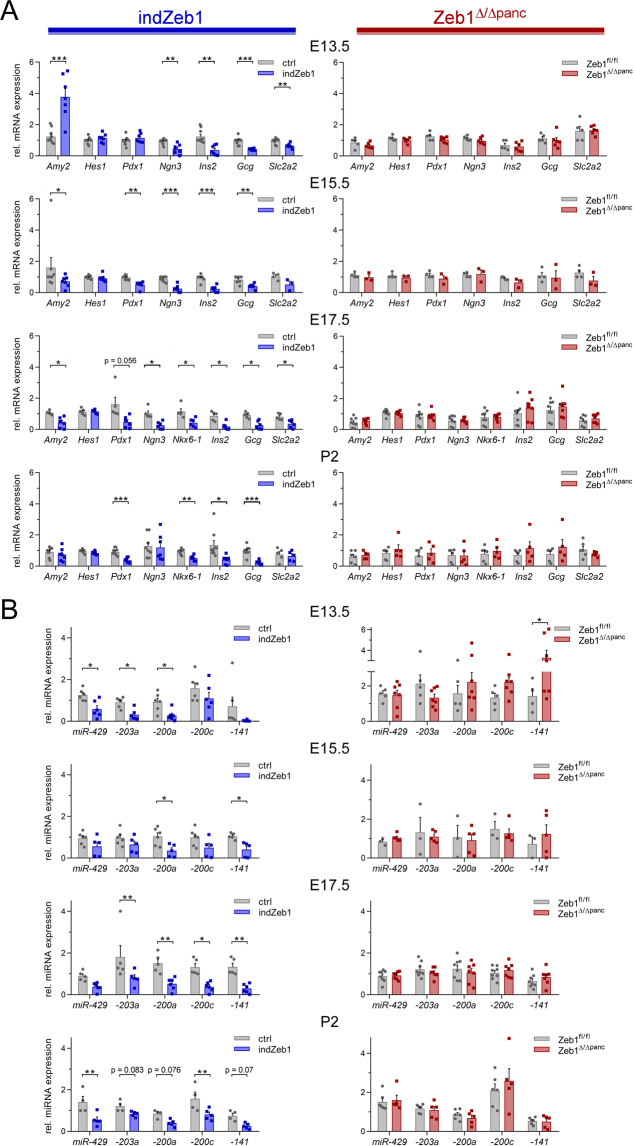
*Zeb1* overexpression causes a dramatic decrease in expression of endocrine marker gene transcripts and affects Zeb1-regulated microRNAs. **A** qRT-PCR analysis of specific lineage markers in pancreas mRNA of indZeb1/ctrl and *Zeb1*^*Δ/Δpanc*^/*Zeb1*^*fl/fl*^ littermates between E13.5 and P2. Consistent reduction of expression was observed in all endocrine-specific genes (*Pdx1*, *Ngn3*, *Nkx6-1*, *Ins2*, *Gcg*, *Slc2a2*) in indZeb1 embryos as well as expression of exocrine-specific *Amy2* is slightly reduced at E15.5 and E17.5, whereas expression of a ductal marker (*Hes1*) is unaffected. *Zeb1*^Δ/Δ*panc*^ embryos do not show robust alterations in gene expression, except for a trending increase in α- and β-cell markers at E17.5 and P2. Note, that *Amy2* expression levels are three orders of magnitude lower at E13.5 than in later stages. *n* = 7–11 (E13.5, indZeb1), *n* = 5 (E13.5, *Zeb1*^Δ/Δ*panc*^); *n* = 7–9 (E15.5, indZeb1), *n* = 3–4 (E15.5, *Zeb1*^Δ/Δ*panc*^); *n* = 5–6 (E17.5, indZeb1), *n* = 7–8 (E17.5, *Zeb1*^Δ/Δ*panc*^); *n* = 7–9 (P2, indZeb1), *n* = 5–6 (P2, *Zeb1*^Δ/Δ*panc*^). **B** qRT-PCR analysis of Zeb1-regulated microRNAs in RNA extracted from the pancreas of indZeb1/ctrl and *Zeb1*^Δ/Δ*panc*^/*Zeb1*^*fl/fl*^ littermates at E17.5 and P2. All analyzed microRNAs show a significant reduction in indZeb1 specimens in at least one stage, whereas no clear difference is observed in *Zeb1*^Δ/Δ*panc*^ miRNA levels compared to controls. *n* = 5–6 (E17.5, indZeb1), *n* = 7–8 (E17.5, *Zeb1*^Δ/Δ*panc*^); *n* = 7–9 (P2, indZeb1), *n* = 5–6 (P2, *Zeb1*^Δ/Δ*panc*^). Statistical significance was determined by two-way ANOVA or Student’s *t*-test and indicated if significance was reached. ***p = 0.0004, *p* = 0.0001; **p = 0.0023, *p* = 0.0014, *p* < 0.005 (mRNA, E13.5); ***p = 0.0002, *p* = 0.0002; **p = 0.0013, *p* = 0.0072; *p = 0.019 (mRNA, E15.5); *p = 0.021, *p* = 0.012, *p* = 0.027, *p* = 0.013, *p* = 0.012, *p* = 0.028 (mRNA, E17.5); ***p = 0.0047; ***p = 0.0004, *p* = 0.0002; **p = 0.0010; *p = 0.014 (mRNA, P2); *p = 0.026, *p* = 0.009, *p* = 0.035, *p* = 0.041 (miRNA, E13.5); *p = 0.020, *p* = 0.038 (miRNA, E15.5); **p = 0.009, *p* = 0.007, *p* = 0.004; *p = 0.011 (miRNA, E17.5); **p = 0.0014, *p* = 0.0036 (miRNA, P2).

We analyzed transcript levels of marker genes in the pancreas of indZeb1 and *Zeb1*^Δ/Δ*panc*^ embryos and pups. Ductal marker gene expression (*Hes1*) was almost unchanged in all embryonic stages and genotypes. *Amy2* (acini-specific) was initially increased, but reduced in indZeb1 mutants between E15.5 and E17.5 (Fig. 7A, left). Substantial changes were observed in markers of endocrine cells of indZeb1 embryos/pups at all analyzed stages. *Pdx1*, *Ngn3* (endocrine progenitors), *Ins2*, *Gcg* (β- and α-cells, respectively), and Glut2 (*Slc2a2*, β-cells) were consistently reduced to 10–50% of control levels, further supporting the finding of a decisive impact of *Zeb1* overexpression on the endocrine lineage (Fig. 7A, left). *Zeb1*^Δ/Δ*panc*^ pancreata showed a trending increase in the expression of the β- and α-cell marker genes *Ins2* and *Gcg* at E17.5 and P2 concomitant to decrease in expression of *Slc2a2* at E15.5 and P2 (Fig. 7A, right). This finding was in agreement with an increase in islet mass in *Zeb1*^Δ/Δ*panc*^ adult mice.

miRNAs play a crucial role in pancreas organogenesis and homeostasis^44^. In particular, the miR-200 family is important and forms a double-negative feedback loop with Zeb1. High miR-200 levels downregulate Zeb1, whereas increased Zeb1 expression represses miR-200s expression affecting cellular differentiation^45,46^. In the pancreas miR-200s are crucial for proper organ formation, but ectopic elevation results in islet β-cell apoptosis in type 2 diabetes mice whereas depletion protects against diabetic loss of β-cells^47,48^. In accordance with Zeb1-mediated repression of miR-200s we found constantly reduced expression of miR-429, miR-200a/c, and miR-141, as well as of miR-203 in indZeb1, which was most significant at E17.5 and P2 (Fig. 7B, left). Apparently, miR-200 repression did not prevent islet and β-cell loss, indicating additional dominating effects of a Zeb1 increase. Acinar and islet-specific loss of *Zeb1* did not induce detectable alterations in miRNA levels (Fig. 7B, right). However, in adult *Zeb1*^Δ/Δ*panc*^ mice, when islet mass was increased, we observed a slight elevation of miR-200s, reaching significance only for miR-200a (Suppl. Fig. S5).

These results confirm that manipulation of *Zeb1* expression mainly affects cells of the endocrine lineage and altered endocrine marker gene expression likely represents a shift in cell composition of the different lineages. Activation and loss of *Zeb1* affects Zeb1-targeted miRNAs, but alterations in miR-200s cannot fully account for the dramatic changes in pancreas morphogenesis and function when Zeb1 is artificially increased or depleted.

## Discussion

Tissue integrity and organ function of the pancreas is dependent on a large variety of signaling cues, transcription factors and metabolic crosstalk. A delicate equilibrium of constant glucose levels in the blood is necessary for homeostasis. This is regulated by ancient mechanisms to cope with periods of poor and exuberant nutrition and physical activity. Constant excess nutrition promotes hyperglycemia, obesity, and type 2 diabetes^1,2^. Transcription factor networks orchestrated by Pdx1, Ptf1α, Sox9, Hnf1α, Gata4/6, Ngn3 are indispensable for morphogenesis and homeostasis of the pancreas and loss of any of these result in a moribund phenotype caused by hypoplasia of islets^1,5,6,12,49^. Our analyses demonstrated that pancreas organogenesis and function is also dependent on Zeb1, a transcription factor mainly confined to non-epithelial cells and a potent inducer of EMT. We found that Zeb1 is expressed at low levels in the pancreas primordium and in acinar and islet cells. Inactivation of *Zeb1* increases islet mass, levels of glucose and Insulin and promotes liver steatosis. In contrast, a ~2-fold elevated Zeb1 expression during embryogenesis blocks proper differentiation and cell survival of acinar and islet progenitors during the second transition leading to pancreas agenesis and postnatal lethality. We show that *Zeb1* expression needs to be tightly regulated for proper pancreas morphogenesis and organ function.

During development EMT-TFs are important to drive key morphogenetic events^26,28^. While Snail induces EMT during gastrulation, Zeb1 is needed for mesoderm-derived differentiation processes at later stages, e.g., of bone, muscle, and melanocytes^23,31–34^. The function of EMT-TFs in normal epithelia might have been largely overlooked due to their very prominent expression in mesoderm and neurectoderm-derived cell types. In the intestine Snail regulates differentiation and homeostasis, since epithelial *Snai1* depletion and overexpression result in apoptotic loss and increase of crypt base columnar cells, respectively^50^. Slug regulates stem/progenitor cells and luminal differentiation in the epithelium during mammary gland morphogenesis^51^. We add another level of EMT-TF function in epithelia with a novel role of Zeb1 in the pancreas that requires tight regulation of expression. Of note, other EMT-TFs are also involved in pancreas formation. Ngn3 increases *Snai1* and *Snai2* (Slug) expression post-transcriptionally in duct cells, which is crucial for efficient *Cdh1* downregulation and delamination of Ngn3+ endocrine progenitors^20,50,52^. In our experiments, we did not detect a contribution of Zeb1 in this process. Although experimental *Zeb1* activation resulted in decreased E-cad expression in the early pancreas, the reduction of all endocrine markers including Ngn3 rather suggests inhibition of endocrine specification by Zeb1. It implies that the role of Zeb1 in specification, differentiation, proliferation, and survival of progenitor cells is intricate and clearly differs from the functions of Snail and Slug. This is in agreement with the non-redundant functions of EMT-TFs in other contexts^23^.

Among several important miRNAs the miR-200 family is a powerful regulator of pancreas function^44^. Members of this family are upregulated during diabetes and drive β-cell apoptosis, whereas hyperglycemia and β-cell apoptosis can be inhibited by ablation of miR-200s^47^. In a rat cell culture model of β-cells (INS-1), knockdown of *Zeb1* was sufficient to induce apoptosis by upregulated miR-200s^48^. In our in vivo analyses we observed a similar anti-correlated relationship leading to miR-200s downregulation when Zeb1 was experimentally increased. However, we neither observed a miR-200 mediated increase in apoptosis upon *Zeb1* knockout nor reduced cell death upon *Zeb1* overexpression, but rather opposite effects. This indicates that the role of Zeb1 and miRNAs in the pancreas is more complex, maybe stage-specific and different in acini and islets. Spatiotemporal differences in activities of the rat insulin^47^ and mouse *Pdx1* promoters to drive gene deletion/activation may also contribute to opposing findings.

Why does a moderate overexpression of *Zeb1* result in such a strong pancreas hypoplasia? Interestingly, *Zeb1* overexpression during hematopoiesis or widespread *Snai1* and *Snai2* overexpression in mice that includes the pancreas did not show such dramatic effects^42,50,53,54^. In contrast to depletion of *Pdx1*, *Ptf1a* or *Mnx1*, which shows early defects in initial pancreatic bud formation or expansion^7,9,10,55^, we here observed no effect on bud formation but on specification of exocrine, endocrine, and duct lineages later due to impaired cell survival and proliferation resulting in hyperplasia. Although we observed major downregulation of endocrine-specific genes—an indication that endocrine cells are more severely affected— our phenotype is different from other loss- and gain-of function mutations. For example, *Ngn3*^*−/−*^, *Neurod1*^*−/−*^, *Nkx2-2*^*−/−*^ and *Nkx6-1*^*−/−*^ mice display reduced or absent endocrine lineages, β-cell differentiation, and/or reduction in islet size^43,56–58^, e.g., due to endocrine to acinar fate switch^16^. The fact that Zeb1 affects cell survival and proliferation of all pancreas lineages is in agreement with its capacity to completely reprogram cells during EMT in cancer, leading to ample changes in cellular properties including metabolism and cell architecture^22,27^. Consequently, these findings support the hypothesis that *Zeb1* expression is required for specific functions, but needs to be tightly regulated and thus kept at a low level for proper organ function.

Interestingly, *Zeb1* deficiency resulted in pronounced liver steatosis indicating systemic consequences of pancreas-specific *Zeb1* depletion. How Zeb1 is integrated in the complex regulation of diabetes, sex steroids, fatty liver, increase of visceral fat and pancreas steatosis still remains obscure. Obesity, hyperglycemia, insulin resistance, and GTT correlate with liver steatosis^59–62^. Pancreas dysfunction during diabetes stimulates peripheral lipolysis, the release of free fatty acids, and deposition in the liver which also correlates with pancreas lipomatosis^63–66^. Moreover, insulin resistance is increasing the portal flux of fatty acids facilitating adipokine release by pancreas adipocytes, which fosters free fatty acids in the circulation and liver steatosis^65,66^. In line with this, our mice that show steatosis in the pancreas but not in the liver might display the priming of this vicious cycle, whereas the presence of a fatty liver in obese diabetic mice indicates a more progressed disease state. Despite the small sample size in our experiments, it is tempting to speculate that Zeb1 attenuates disease pathology by inhibiting pancreas lipid deposition and liver steatosis. To test this hypothesis a more detailed analysis is required.

In conclusion, our findings indicate that Zeb1 function in the pancreas is indispensable for proper morphogenesis and homeostasis requiring very tight locus control to maintain low Zeb1 expression levels.

## Materials and methods

### Mice

Animal husbandry and all experiments were performed according to the European Animal Welfare laws and guidelines. The protocols were approved by the committee on ethics of animal experiments of Bavaria, Germany (Regierung von Unterfranken, Würzburg; TS-18/14 and 55.2-DMS-2532-2-59). Power analysis was used to calculate the sample size required for animal experiments. Animals were kept on a 12:12 h light-dark cycle and provided with food and water ad libitum in the animal facility of the Friedrich-Alexander University of Erlangen-Nürnberg. The *Pdx1*-Cre transgene (Tg(Pdx1-cre)6Tuv), generation of the conditional *Zeb1* knockout alleles, *Zeb1*^*fl*^ (Zeb1^tm1.1Mpst^) and *Zeb1*^*del*^ (Zeb1^tm1.2Mpst^) as well as the mTmG Cre reporter (Gt(ROSA)26Sor^tm4(ACTB-tdTomato,-EGFP)Luo^) have been described previously^29,36,37^. Coding sequences of HA-tagged Zeb1 were inserted into the *Rosa26* locus for Cre-mediated gene activation^42^. A detailed description of *R26-Zeb1*^*tg*^ mice will be described elsewhere. All mice were kept on a C57BL/6N background. Mice were sacrificed at indicated timepoints and tissues were fixed in 4% paraformaldehyde (PFA)/PBS overnight and embedded into paraffin, fresh frozen in TissueTek OCT at −80 °C or subjected to RNA isolation.

### Induction of type 2 diabetes and CP

For induction of type 2 diabetes (T2D) mice were switched from normal chow diet (ND) to HFD with 60 kJ % fat (Ssniff, Soest, Germany) directly after weaning for 19–20 weeks^40^. Mice under HFD and control ND were weighed and venous blood glucose levels measured at indicated time-points to assess T2D progression.

CP was induced by hydrodynamic gene delivery of an *Il17a* expression vector into the liver, using a mouse *Albumin* promoter/*Afp* enhancer sequence of pLIVE (Mirus, Madison, WI, USA) to drive mouse *Il17a* expression^41^.

### GTT and measurement of blood glucose and insulin levels

For the intraperitoneal GTT mice were fasted for 4–6 h and injected intraperitoneally with 2 g/kg body weight glucose^38,40^. Venous blood samples were taken before and during GTT at indicated time-points and blood glucose concentrations were measured with a glucometer (VPD, Bled, Slovenia). For quantification of insulin levels, plasma was isolated from the supernatant after centrifugation of blood samples for 15 min at 2000 × *g* at 4 °C. Quantification was carried out by the Ultra Sensitive Mouse Insulin ELISA Kit (Crystal Chem, Zaandam, Netherlands) according to the manufacturer’s instructions.

### Statistical analysis

Prism 8 (GraphPad, San Diego, CA, USA) was used for statistical analysis. Data are represented by means ± SEM of n biological replicates as indicated. One-way ANOVA with Tukey multiple comparisons test was used to analyze ratios of pancreas weight versus total body weight, for quantification of Insulin/Glut2-areas and for proliferation and apoptosis. Two-way ANOVA with Tukey multiple comparisons test or Student’s *t*-test was used for qRT-PCR data for *Zeb1*^*fl/fl*^, *Zeb1*^*+/*Δ*panc*^, and *Zeb1*^Δ/Δ*panc*^. qRT-PCR data between E13.5 and P2, proliferation and apoptosis, GTT, glucose and Insulin levels, liver weight and steatosis were analyzed by Student’s multiple *t*-test, corrected for multiple comparisons, and the Holm-Sidak method for correction. Survival of indZeb1 was tested for significance by a log-rank (Mantel-Cox) test. In all analyses two-tailed tests were performed. *P*-values of statistical significance are represented as: *p < 0.05, **p < 0.01, ***p < 0.001. The variance between the statistically compared groups are similar. No estimate of variation has been performed within each group of data prior to statistical analysis.

## Supplementary information

Supplementary material
