# Supplementary material for: Pancreas morphogenesis and homeostasis depends on tightly regulated Zeb1 levels in epithelial cells
Source: Cell Death Discov. 2021 Jun 11;7:138. doi: 10.1038/s41420-021-00522-z (PMC8192546; doi:10.1038/s41420-021-00522-z)
Supplement: Supplementary file 1 — Supplementary material [file 41420_2021_522_MOESM1_ESM.pdf]

Supplementary material and methods

Histology and immunohistochemistry (IHC)

PFA-fixed tissues were embedded into paraffin and sectioned at 4-5 µm using a RM2255 Automated Microtome (Leica Microsystems, Wetzlar, Germany). Haematoxylin/Eosin (HE), Masson's trichrome staining (MTS) and immunohistochemistry (IHC) was carried out as described [1]. The following antibodies were used: CD45 (Thermo Fisher Scientific, Darmstadt, Germany, 14-0451, 1:200), cleaved Caspase 3 (Cell Signaling, Danvers, MA, USA, 9664, 1:200), Glut2 (Santa Cruz, Dallas, TX, USA, sc-518022, 1:200), Insulin (BioGenex, Fremont, CA, USA, MU029-UC, 1:400), Ki67 (Abcam, Cambridge, UK, ab16667, 1:300), Nkx6.1 (R&D Systems, Minneapolis, MN, USA, AF5857, 1:3000), phH3 (Cell Signaling, 9701S, 1:500), Slug (Cell Signaling, 9585, 1:150), Snail (Cell Signaling, 3879, 1:200), Zeb1 (Novus Biologicals, Centennial, CO, USA, NBP1-05987, 1:250), Zeb2 (Novus Biologicals, NBP1-82991, 1:200).

For assessing the islet mass and Ki67 and cleaved Caspase 3-positive cell ratios, two pancreas slides with at least 100 µm distance were used for IHC and to calculate the relative Insulin/Glut2-positive area and Ki67 and cleaved Caspase 3-positive epithelial cells from three different 500 $µm^2$ from each slide. Liver steatosis was assessed from two HE stained sections with 100 µm distance and the affected area was estimated over the total liver area.

Cryosectioning, oil red O staining (ORO) and immunofluorescence staining

For cryosectioning 3-5 mm tissue specimens were either fresh frozen or 2-h PFA-fixed, followed by a 4-h/over-night incubation in 15%/30% sucrose/PBS before embedding into TissueTek OCT (Sakura, Staufen, Germany) and sectioned at 8-10 µm using a CM3050 S Research Cryostat (Leica). Lipid content in cryosections was visualized by oil red o staining (ORO). Cryosections were fixed in 4% PFA/PBS for 10 min and washed in tap water. Subsequently, they were briefly immersed in 60% isopropanol and stained with ORO working solution (Sigma Aldrich, Munich, Germany, O0625) for 15 min, followed by rinsing in 60% isopropanol and briefly immersed in Mayer's Hematoxylin.

For indirect immunofluorescence labeling cryosections were fixed in 4% PFA/PBS for 10 min, then permeabilized for 10 min in 0.25% Triton-X100/PBS. After blocking with 3% BSA/PBS for 30 min, slides were incubated with appropriate antibody solutions over-night at 4°C followed by incubation with alexa488, alexa555 and CF604R-conjugated secondary

antibodies (Sigma Aldrich) for one hour at RT. All images were acquired on a DM5500B microscope using the LAX Software (Leica). For pseudo-overlay, images from consecutive sections individually stained for the indicated antibodies and co-stained with anti-E-cadherin, were used. An approximate overlay of the three markers was generated by aligning the E-cadherin staining of the individual consecutive sections, and then displaying only the indicated markers. Detection of GFP/Tomato derived from the mTmG allele combined with IF was carried out on 2-h fixed sections as described above. Anti-Zeb1 staining was performed as described above in the dark using a CF604R-conjugated secondary antibody (Sigma Aldrich). The following antibodies were used: E-Cadherin (BD Transduction Laboratories, San Jose, CA, USA, 610182, 1:200), Glucagon (Abcam, ab92517, 1:500), Pdx1 (Abcam, ab47267, 1:5000), Ptf1α (rabbit polyclonal, kindly provided by Chris Wright, 1:1000) [2], Sox9 (Merck Millipore, Darmstadt, Germany, AB5535, 1:1000), Zeb1 (Bethyl Laboratories, Montgomery, TX , USA, IHC-00419, 1:200).

RNA isolation, cDNA synthesis, qRT-PCR and miRNA quantification

Total RNA was isolated and reversely transcribed using the RNeasy Plus Mini Kit (Qiagen, 74136) and the RevertAid First Strand cDNA Synthesis Kit (Thermo, K1622) for mRNA and the miRCURY LNA RT Kit (Exiqon, Vedbaek, Germany, 203301/Qiagen, Hilden, Germany, 339340) for miRNA. mRNA transcripts were detected by using cDNA corresponding to 7.5 ng total RNA (or 1.5 ng for E13.5 and E15.5 pancreas) with 300 nM gene-specific primers, the Universal Probe Library (Roche, Penzberg, Germany, 04869877001) and the TaqMan Universal Master Mix (Applied Biosystems, Foster City, CA, USA, 4440040) according to the manufacturers' instructions in a 12 µl volume. miRNAs were analyzed with the miRCURY ExiLENT SYBR Green Kit (Exiqon, 203421/Qiagen, 339345) with specific primer sets according to the manufacturer's instructions (Supplementary Tables 1 and 2). All samples were run in a LightCycler 480 II (Roche) and values were normalized to *Gapdh* and *Mir16-1* levels.

References

1. Krebs AM, Mitschke J, Lasierra Losada M, Schmalhofer O, Boerries M, Busch H*, et al.* The EMT-activator Zeb1 is a key factor for cell plasticity and promotes metastasis in pancreatic cancer. *Nat Cell Biol* 2017, **19**(5)**:** 518-529.

2. Hald J, Sprinkel AE, Ray M, Serup P, Wright C, Madsen OD. Generation and characterization of Ptf1a antiserum and localization of Ptf1a in relation to Nkx6.1 and Pdx1 during the earliest stages of mouse pancreas development. *J Histochem Cytochem* 2008, **56**(6)**:** 587-595.

Supplementary tables

Supplementary Table 1: Primer sequences and probes used for mRNA qRT-PCR

Gene	Forward	Reverse	UPL
Actb	5'-AAGGCCAACCGTGAAAAGAT-3'	5'-GTGGTACGACCAGAGGCATAC-3'	#56
Amy2	5'-GTTATCCGCAAGTGGAATGG-3'	5'-CAAACACAAGGGCTCTGTCA-3'	#7
Cdh1	5'-ATCCTCGCCCTGCTGATT-3'	5'-ACCACCGTTCTCCTCCGTA-3'	#18
Gapdh	5'-AGCTTGTCATCAACGGGAAG-3'	5'-TTTGATGTTAGTGGGGTCTCG-3'	#9
Gcg	5'-TACACCTGTTCGCAGCTCAG-3'	5'-TTGCACCAGCATTATAAGCAA-3'	#5
Hes1	5'-TGCCAGCTGATATAATGGAGAA-3'	5'-CCATGATAGGCTTTGATGACTTT	#20
Hprt1	5'-TCCTCCTCAGACCGCTTTT-3'	5'-CCTGGTTCATCATCGCTAATC-3'	#95
Ins2	5'-GAAGTGGAGGACCCACAAGT-3'	5'-CAGTGCCAAGGTCTGAAGGT-3'	#32
Ngn3	5'-AATCGCATGCACAACCTCAACTCG-3'	5'-AGCGCAGGGTCTCGATCTTTGTAA-3'	#42
Nkx6-1	5'-CCCGGAGTGATGCAGAGT-3'	5'-GAACGTGGGTCTGGTGTGTT-3'	#103
Pdx1	5'-GAAATCCACCAAAGCTCACG-3'	5'-CGGGTTCCGCTGTGTAAG-3'	#51
Vim	5'-TGCGCCAGCAGTATGAAA-3'	5'-GCCTCAGAGAGGTCAGCAAA-3'	#79
Zeb1	5'-AGGTGATCCAGCCAAACG-3'	5'-GGTGGCGTGGAGTCAGAG-3'	#93
Zeb2	5'-CCAGAGGAAACAAGGATTTCAG-3'	5'-AGGCCTGACATGTAGTCTTGTG-3'	#42

Supplementary Table 2: Exicon assays used for miRNA qRT-PCR

miRNA	Assay name	Ordering number	Supplier
miR-141	hsa-miR-141-3p	204504	Exicon
miR-16	hsa-miR-16-5p	205702	Exicon
miR-200a	hsa-miR-200a-3p	204707	Exicon
miR-200b	hsa-miR-200b-3p	206071	Exicon
miR-200c	hsa-miR-200c-3p	204482	Exicon
miR-203a	hsa-miR-203a	205914	Exicon
miR-429	mmu-miR-429-3p	205068	Exicon
miR-429	mmu-miR-429-3p	YP00205068	Qiagen

Supplementary figure legends

Supplementary Figure S1. Zeb1 is expressed in epithelial cells of the developing pancreas. (A) Top, schematic representation of the *Zeb1*^{del} locus in homozygous zygotic *Zeb1* knockout mice. Bottom, immunohistochemical analysis of Zeb1 on sagittal sections of *Zeb1*$^{+/+}$ and *Zeb1*$^{del/del}$ embryos at E11.5 (left) and E17.5 (right) in the pancreas and other Zeb1 expressing tissues. Weak, but specific Zeb1 detection in epithelial cells of the pancreas is observed that was lost in *Zeb1*-deficient embryos. nt, neural tube; panc, pancreas; si, small intestine; sto, stomach. Scale bar, 100 µm. (B) Top, schematic representation of alleles used for detection of Cre-mediated recombination in the pancreas by *Pdx1*-Cre. The mTmG reporter allele provides membrane-bound tdTomato (Tom) expression in unrecombined cells and GFP expression in cells with efficient Cre activity and their descendants. Bottom, mice with (*Cre*+;*Zeb1*$^{+/+}$) and without the *Pdx1*-Cre allele (*Cre*-;*Zeb1*$^{+/+}$) are shown. Simultaneously, *Zeb1* depletion was analyzed in compound mice with an additional homozygous *Zeb1*$^{flox/flox}$ configuration (*Cre*+;*Zeb1*$^{fl/fl}$). Tomato and GFP autofluorescence detection and indirect immunofluorescence staining of Zeb1 on adult pancreas cryosections. Representative images are shown to demonstrate moderate to highly efficient Cre-mediated recombination and *Zeb1* depletion. Boxed area labels region of the Zeb1-only channel (magenta) with higher magnification. Open arrowheads point to Zeb1+ epithelial cells. Scale bars, 100 µm (regular) and 20 µm (inset).

Supplementary Figure S2. *Zeb1* depletion results in increased lipid accumulation in the pancreas and more severe liver steatosis during challenging the endocrine pancreas in a sex dependent manner. (A) Summary of blood glucose (left) and insulin (right) levels of all mice confirming significantly reduced glucose and not yet significantly reduced insulin levels in *Zeb1*$^{\Delta/\Delta panc}$ mice. (B) Body weight in diabetic mice at the end of HFD

treatment indicates that males are more susceptible to gain weight in both genotypes. n=12 (HFD total), n=4 (ND total), n=5 (HFD males), n=1 (ND males), n=7 (HFD females), n=3 (ND females). (C-E) Absolute blood glucose (C) and insulin levels (E) at the end of HFD and ND-fed littermates, separated into males and females and longitudinal blood glucose measurement during HFD (D). Note, that steady-state blood glucose levels in HFD and ND-fed mice are constantly increasing during HFD, but tending to be lower in *Zeb1*$^{\Delta/\Delta panc}$ individuals. A 4-hour fasting period at the end of the HFD results in unchanged to moderately increasing glucose levels in *Zeb1*$^{\Delta/\Delta panc}$ mice, whereas they decrease in *Zeb1*$^{fl/fl}$ mice (D). n=3 (HFD males), n=1 (ND males), n=3 (HFD females), n=1 (ND females) (C, E); n=6 (HFD), n=2 (ND) (D). (F) Absolute blood glucose levels during intraperitoneal GTT after HFD. n=9 (HFD), n=4 (ND). (G, H) Weight changes of liver (G), pancreas and spleen (H) during HFD, separated into males and females of *Zeb1*$^{\Delta/\Delta panc}$ and *Zeb1*$^{fl/fl}$ mice. n=6 (HFD), n=2 (ND), n=3 (HFD males), n=1 (ND males), n=3 (HFD females), n=1 (ND females). (I) Quantification of lipid content in pancreas by ORO and liver steatosis by histological assessment, demonstrating that *Zeb1*$^{\Delta/\Delta panc}$ males preferentially show liver steatosis, whereas *Zeb1*$^{\Delta/\Delta panc}$ females show pancreas lipid deposition. n=3 (HFD males), n=1 (ND males), n=3 (HFD females), n=1 (ND females). Statistical significance was determined by Student's t-test and indicated if significance was reached. *, p=0.017.

Supplementary Figure S3. Tissue damage and inflammation is unchanged upon challenging the exocrine pancreas by induction of chronic pancreatitis in *Zeb1*$^{\Delta/\Delta panc}$ mice. Acute phase of chronic pancreatitis, induced by hepatocyte-specific expression of a *Il17a* cDNA expression (IL17) or empty control vector (ctrl). Mice were sacrificed 15 days after hydrodynamic gene delivery. Histological analysis by HE (top panel), Masson's trichrome staining (MTS, lower panel) and anti-CD45 immunohistochemistry (middle panel) of pancreas paraffin sections shows severe tissue damage in IL17a individuals that is combined by substantial immune cell infiltration and moderate collagen deposition and fibrosis. Note, that *Zeb1*$^{\Delta/\Delta panc}$ and *Zeb1*$^{fl/fl}$ mice show no difference in IL17a-mediated chronic

pancreatitis. Open arrows indicate individual tissue resident CD45+ hematopoietic cells. Scale bar, 100 µm.

Supplementary Figure S4. Detailed analysis of lineage specification markers of indZeb1 mice at E15.5. (A) Two examples of each genotype were cryosectioned and consecutive sections were subjected to immunofluorescence labeling as indicated, with co-labeling of E-cad (green) and nuclei with DAPI (blue). Single channel images of the perspective lineage marker and overlays are shown at low (left) and high magnifications (right). Sections are oriented with anterior to the top and dorsal to the right. Scale bar, 100 µm. (B) Images of individual sections stained for markers as indicated in grayscale and used for the pseudo-overlay in Fig. 6. Sections are oriented with anterior to the top and dorsal to the right. Scale bar, 100 µm.

Supplementary Figure S5. Zeb1 depletion induces upregulation of miR-200s in the adult pancreas. qRT-PCR analysis of Zeb1-regulated microRNAs in RNA extracted from pancreas of indZeb1/ctrl and $Zeb1^{\Delta/\Delta panc}$/$Zeb1^{fl/fl}$ littermates at 6 months of age. miR-200a and miR-141 show increased expression in $Zeb1^{\Delta/\Delta panc}$ samples. n=2-3.

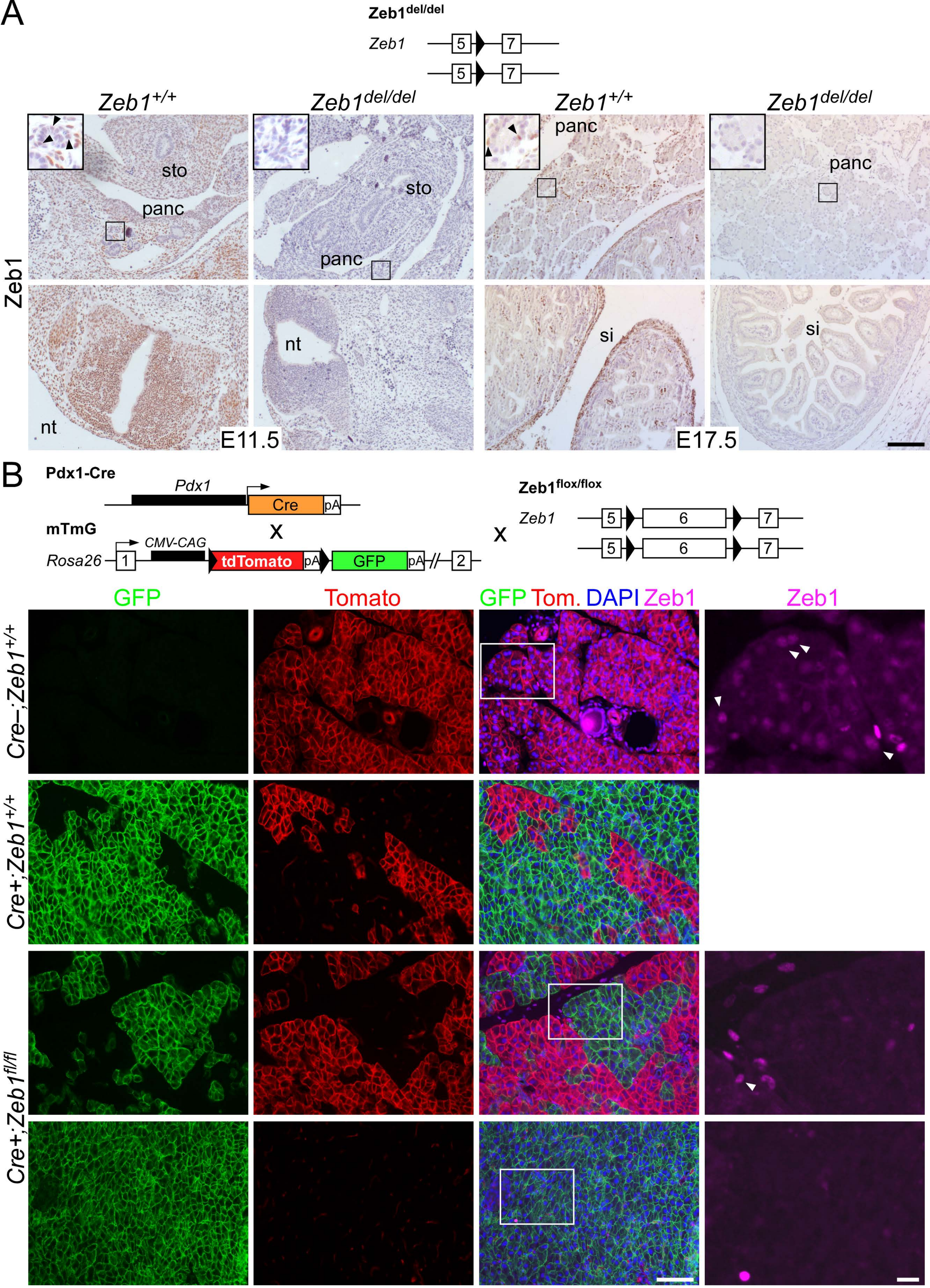

Lasierra_Supplementary Figure S1

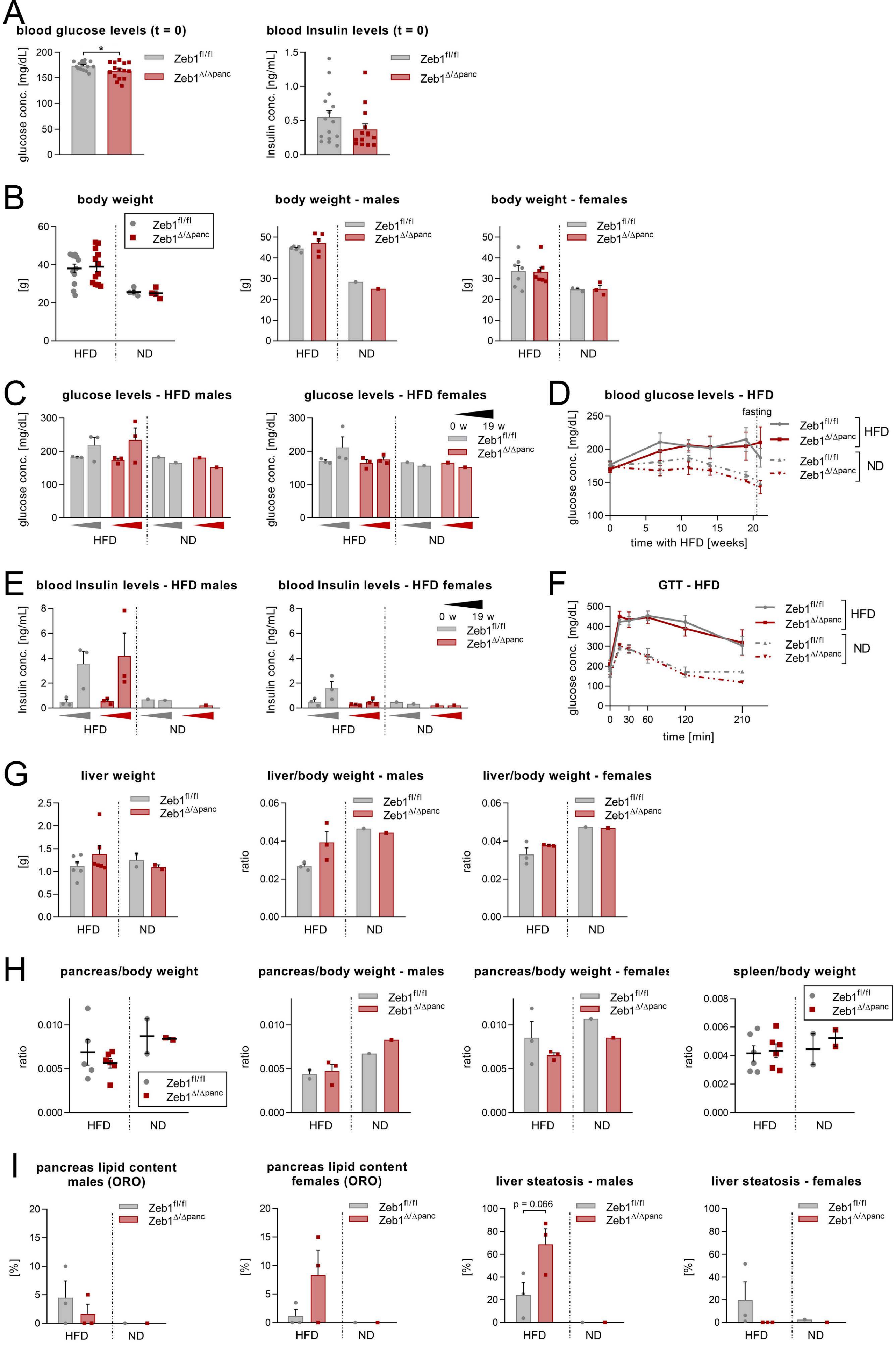

Lasierra_Supplementary Figure S2

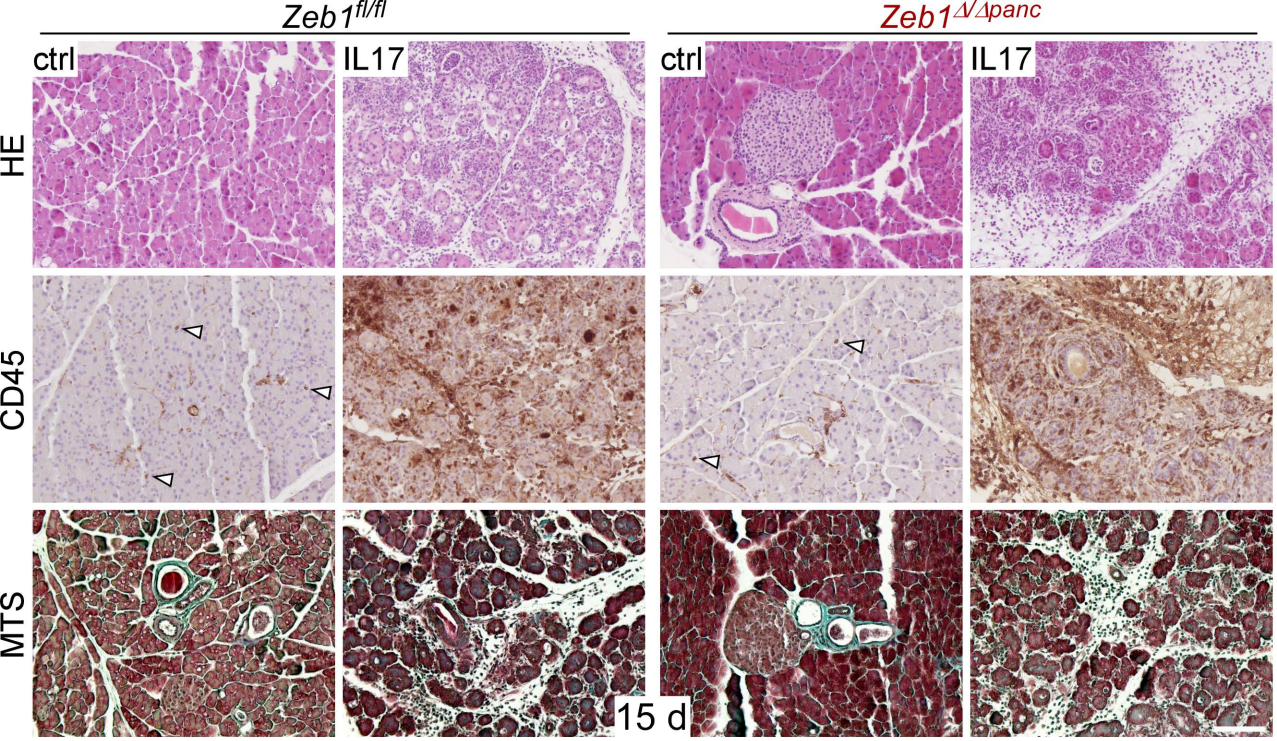

Lasierra_Supplementary Figure S3

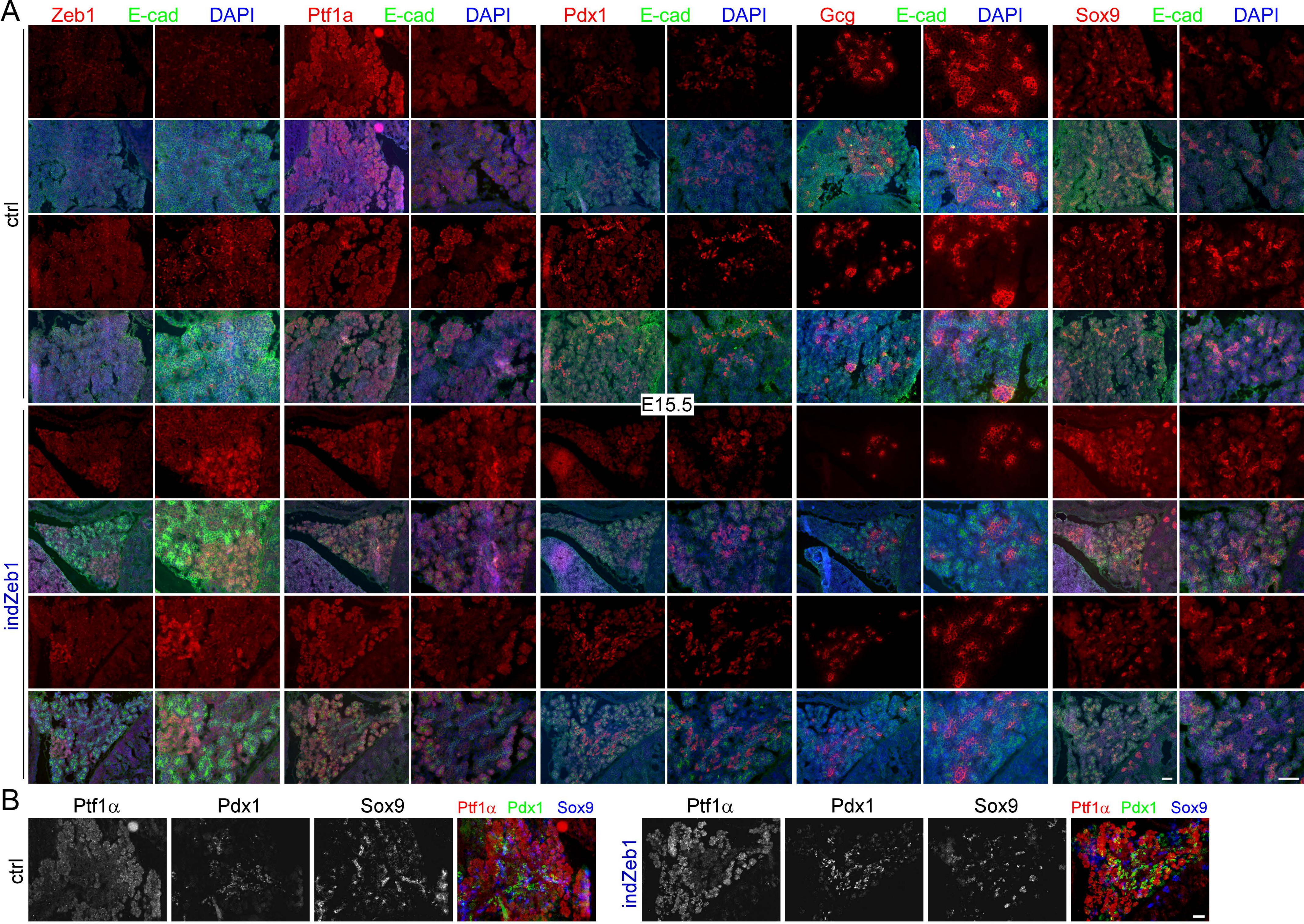

Lasierra_Supplementary Figure S4

miRNA levels in adult pancreas (6 months)

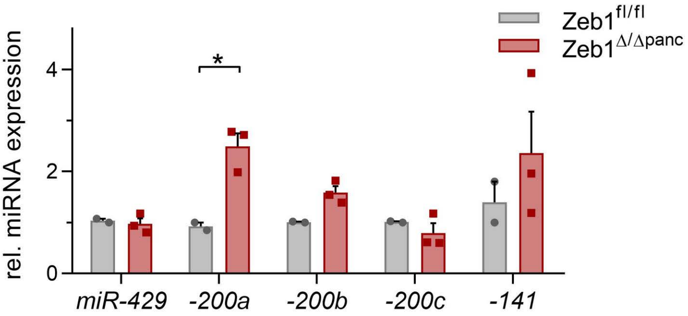

Lasierra_Supplementary Figure S5
